# Evaluation of physicians’ knowledge of deprescribing, deprescribing tools and assessment of factors affecting deprescribing process

**DOI:** 10.1186/s12875-023-01990-1

**Published:** 2023-01-25

**Authors:** Wuraola Akande-Sholabi, Comfort O. Ajilore, Temitope Ilori

**Affiliations:** 1 Department of Clinical Pharmacy and Pharmacy Administration, Faculty of Pharmacy, University of Ibadan, Ibadan, Nigeria; 2 Department of Community Medicine, College of Medicine, University of Ibadan, Ibadan, Nigeria; 3 Department of Family Medicine, University College Hospital, Ibadan, Nigeria

**Keywords:** Deprescribing, Polypharmacy, Beers Criteria, Older adults

## Abstract

**Background:**

Polypharmacy is a common global health concern in the older population. Deprescribing has been acknowledged as an important aspect of medication use review that helps to reduce polypharmacy, inappropriate medication uses and medication adverse events, thus ensuring medication optimization and improving health-related quality of life. As physicians are primarily responsible for prescribing and monitoring of drug therapy, their perception of deprescribing and knowledge of available deprescribing tools is highly important. This study aimed to explore physicians’ knowledge of deprescribing, deprescribing tools and factors that may affect the deprescribing process.

**Methods:**

This was a cross-sectional survey carried out among 70 physicians in selected units of a teaching hospital in Nigeria between May and June 2022. Social-demographic information, knowledge of deprescribing and deprescribing tools were obtained using a self-administered, semi-structured questionnaire, while barriers and enablers of medication deprescribing were assessed with modified Revised Patients’ Attitudes Towards Deprescribing (rPATD) Questionnaire. Descriptive and bivariate analyses were carried out using SPSS and α was set at *p* < 0.05.

**Results:**

Most of the physicians (56; 80.0%) were aware of the term “deprescribing” and had good knowledge (53; 75.7%) of the steps to deprescribing. However, (16; 22.9%) respondents knew of the deprescribing tools, of this, (5; 31.3%) were aware of Beers criteria and STOPP/START criteria. Awareness of the term “deprescribing” was significantly associated with knowledge of deprescribing steps (*p* = 0.012), while knowledge of deprescribing tools was significantly associated with; awareness of the term “deprescribing” (*p* = 0.029), and daily encounters with older multimorbid patients (*p* = 0.031). Very important factor affecting physicians deprescribing decisions include benefit of the medication. The most common barrier is lack of information for a full clinical picture of the patient.

**Conclusion:**

The physicians had good knowledge of the term “deprescribing” and the steps to deprescribing. Specific measures to target the barriers faced by the physicians in deprescribing medications and policies to implement physicians use of existing guidelines to facilitate their deprescribing decisions are essential.

## Background

As a result of aging, older adults suffer more illnesses than younger adults including chronic conditions, resulting in the concurrent use of multiple medicines (polypharmacy) for the treatment and management of these respective conditions [1]. The benefits and risks are not clear or are unavailable for older, medically complex adults who are usually not well represented in research [2, 3].

Health system in Nigeria is overburdened with a doctor-patient ratio greater than the WHO recommendation, 1: 5000 versus 1:600, leading to an overall poor patient compliance with medical advice and resultant negative health outcomes [3]. Nigeria has a relatively young population with the elderly accounting for only 4.3% of the national population, (National population commission, 2009) [4]. However, In Nigeria, roughly 25 to 35% of older persons aged 60 years or older, experience polypharmacy [5]. Polypharmacy has been associated with an increase in overall patient burden, increased risk of adverse events, medication non-adherence, and most importantly, increased use of potentially inappropriate medications (PIMs) which are medicines whose risk supersedes their intended benefits [6, 7]. PIMs use in the older adults is a public health concern, associated with increased morbidity, mortality, and poor health-related quality of life with a prevalence range of 25.5% to 57.6% in Nigeria [5, 8, 9].

Deprescribing refers to the planned, systematic reduction or removal of medications that are inappropriate or unnecessary, supervised by a health care professional to optimize health care by reducing medication burden and risk, and improving overall health outcomes [10]. Although deprescribing is a component of prescribing, in practice, it is not routinely practiced as evidenced by the high prevalence of polypharmacy and PIM use in the elderly [11, 12]. Scott et al. have outlined steps involved in carrying out the deprescribing process; and several tools/guidelines exist to facilitate deprescribing in the elderly including Beers criteria, STOPP/START criteria, Medication Appropriateness Index, and Good Palliative-Geriatric Practice algorithm [13–16].

Deprescribing is complicated by several patients and physician-related factors which may be counted as either enablers or barriers to the deprescribing process. These include; the physician’s self-efficacy, patient’s desires, and physician’s knowledge and awareness of deprescribing tools [17–19]. In a systematic review of the burden of polypharmacy and potentially inappropriate medications use in Nigeria, a clarion call for deprescribing was raised after reporting scarcity of data on deprescribing despite the rising levels of polypharmacy and PIMs [3]. As physicians are primarily responsible for deprescribing in Nigeria, it is therefore important to explore the physicians’ knowledge and willingness to endorse appropriate deprescribing as well as the associated enablers and barriers to develop guidelines and measures to optimize appropriate medication use in the older population. Hence, this study team aimed to explore physicians’ knowledge of deprescribing, deprescribing tools and factors that may affect the deprescribing process.

## Methods

### Study design

This study is a cross-sectional questionnaire-based survey carried out among physicians in selected units at a teaching hospital in Nigeria, between the periods from May to July 2022.

### Study setting

The study was carried out in the departments of Family medicine, Medicine and Psychiatry in University College Hospital Ibadan. The teaching hospital is a 900-bed tertiary healthcare institution in Nigeria, affiliated with a university. It is a foremost site for undergraduate and post-graduate residency training for physicians, as well as clinical training for other categories of healthcare professionals including pharmacists, nurses, and medical laboratory scientists.

### Study population

Participants for the study were selected from the departments of Family medicine, Medicine, and psychiatry.

### Inclusion and exclusion criteria

Eligible participants were board certified physicians who gave voluntary consent to participate in the study. Participating physicians must have a minimum of one year of clinical practice experience. House officers, physicians on leave and those who declined participation were excluded.

### Sample size determination

The number of physicians working in these units (Family medicine, Psychiatry, medicine) was obtained from the hospital management board in University College Hospital. Based on the estimated population of 90 registered physicians and using the assumption of a 95% confidence level and a 5% margin of error, a sample size of 73 was obtained using the Yamane sample size determination formula [20]. Adjusting for a 10% non-response rate, a target sample population of approximately 81 was obtained.

### Data collection instrument

The questionnaire used for data collection was designed by the researchers (2 clinical pharmacists and a family medicine physician) after a thorough review of relevant studies and was adjusted to reflect the local perspectives [11, 17, 21–23]. The questionnaire consisted of four sections. Section 1 obtained information on physician background characteristics. Section 2 included semi-structured questions addressing physicians’ awareness and knowledge of deprescribing and deprescribing tools. The deprescribing tools being evaluated for are Beers and STOPP/START criteria[13, 14] for potentially inappropriate medication use in the elderly. Section 3 evaluated factors important to physicians for their deprescribing decisions using a 4-point Likert scale ranging from 1 = “Not Important” to 4 = “Very Important”. Section 4 explored possible enablers and barriers to deprescribing from the physician’s perspectives based on questions from the modified revised Patients’ Attitudes Towards Deprescribing (rPATD) [24] Questionnaire which consist of a 4-point Likert scale ranging from 1 = “Strongly Disagree” to 4 = “Strongly Agree”.

### Pretest and content validation

Content validity of the questionnaires was carried out by a pharmacist in academia. Appropriate modifications to some questions were made, and some were rephrased to avoid ambiguity. Subsequently, the questionnaire was given to five (5) physicians in the University College Hospital, Ibadan, Oyo State. Appropriate modifications were made based on the feedback from these respondents and some ambiguous questions were rephrased. These respondents were excluded from the study. The internal consistency from the pre-test was revealed by a Cronbach alpha value of 0.72.

### Sampling and data collection procedure

Purposive sampling technique was used to enroll physicians for participation. Eligible physicians were approached at their individual work units and the questionnaire was self-administered after consent was obtained. Anonymity and confidentiality of response was assured while participation was entirely voluntary.

### Data analysis

Data entering, cleansing and analysis were done using the Statistical Package for Social Science (SPSS) version 23.0, IBM Corp. Physician background characteristics were examined using descriptive statistics. Factors important for deprescribing decisions, barriers and enablers to deprescribing on the Likert scale were calculated in percentages and represented on a stacked bar chart. Pearson Chi square (χ2) was used to determine the association between background characteristics of physicians and knowledge of deprescribing, and knowledge of deprescribing tools. A threshold of *p*-value < 0.05 is significant.

For this study, knowledge of the deprescribing process was determined using the order of steps cited by Scott et al. [15].

Ethical approval for the study was obtained from the Institutional Ethical Review Board and the Chairman, Medical Advisory Committee, of the teaching hospital.

## Results

A total of 85 questionnaires were given out, and 70 were filled and returned giving a response rate of 82%.

Background information of participating physicians is shown in Table 1. Females were 52.9% and the mean years of clinical experience was 9 years. A total of 52.9% of the physicians had fifteen or more daily consultations, and 8.6% reported daily encounters with older multimorbid patients. However, only 20.0% reported daily encounters with multimorbid older patients having polypharmacy. Of the 70 physicians, 22.9% reported they deprescribe daily during consultations.

**Table 1:** Physician characteristics and Consultation Pattern (*n* = 70)

Variables	Frequency (%)
**Gender**	
Male	33 (47.1)
Female	37 (52.9)
**Years of clinical experience**	
1–5	24( 34.3)
6 -10	30(42.9)
11–15	7(10.0)
> 15	9(12.9)
**Number of daily consultations**	
< 15	33 (47.1)
15–25	28 ( 40.0)
26–35	7 (10.0
> 35	2 (2.9)
**Frequency of attending to patients aged 60 years and above with multimorbidity daily**	
Never	0 (0.0)
Few times monthly	22 (31.4)
< than 3 days weekly	10 (14.3)
> 3 days weekly but not daily	18 (25.7)
Daily	20 (28.6)
**Frequency of attending to patients aged 60 years and above with polypharmacy (> 5 medications) daily**	
Never	4 (5.7)
Few times monthly	21 (30.0)
< 3 days weekly	18 ( 25.7)
> 3 days weekly but not daily	13 (18.6)
Daily	14 (20.0)
**Frequency of deprescribing during daily consultation**	
Never	8 (11.4)
Few times monthly	26(37.1)
< than 3 days weekly	13(18.6)
> 3 days weekly but not daily	7(10.0)
Daily	16(22.9)

The majority of the total physician population (80.0%) were aware of the term “deprescribing” and 75.7% of the total population had good knowledge of the deprescribing steps as described by Scott et al.^17^ Only 21.4% of the total physician population has had any previous training on deprescribing and several sources of training were cited. Overall, 22.9% of physicians reported knowledge of at least one deprescribing tool/guideline with Beers criteria and STOPP/START criteria cited the most. See Table 2.

**Table 2:** Physician awareness, knowledge, and training on deprescribing and deprescribing tools (*n* = 70)

Variables	Frequency (%)
**Awareness of the term “deprescribing**”	
Yes	56 ( 80.0)
No	14 ( 20.0)
**Knowledge of order of deprescribing steps**	
Good knowledge	53 (75.7)
Poor knowledge	17 (24.3)
**Previous training on deprescribing**	
Yes	15 (21.4)
No	55 (78.6)
**# Place/sources of previous training on deprescribing (*n* = 22)**	
Geriatric courses	1 (4.5)
Online course	3 (13.6)
Personal study	8 (36.4)
Postgraduate update courses/ training	4 (18.2)
Seminars	5 (22.7)
Undergraduate course	1 (4.5)
**Awareness of deprescribing tools**	
Yes	16 (22.9)
No	54 ( 77.1)
**#List of known deprescribing tools ( *n* = 16)**	
Beers’ criteria	5 (31.3)
STOPP /START	5 (31.3)
Beers criteria and STOPP /START	6 (37.5)
**Frequency of use of specific deprescribing tools**	
**Beers criteria**	
Often use	3 (4.3)
Sometime use	5 ( 7.1)
Rarely use	2 (2.9)
Know of but never use	1 (1.4)
Never heard of	59 (84.2)
**STOPP/START criteria**	
Often use	3 (4.3)
Sometime use	3 (4.3)
Rarely use	2 (2.9)
Know of but never use	3 (4.3)
Never heard of	59 (84.2)

# = multiple response variable

Awareness of the term “deprescribing” was significantly associated with knowledge of deprescribing steps (*p* = 0.012), while knowledge of deprescribing tools was significantly associated with; awareness of the term “deprescribing” (*p* = 0.029), daily encounters with older multimorbid patients (*p* = 0.031), and previous training on deprescribing (*p* = 0.000) Table 3.

**Table 3:** Association between socio-demographic variables and knowledge of deprescribing steps

Variables	Knowledge of deprescribing steps		Knowledge of deprescribing tools	
	Good knowledge Frequency (%)	Poor knowledge Frequency (%)	Yes Frequency (%)	No Frequency (%)
**Gender**				
Male (*n* = 33)	24 (72.7)	9 (27.3)	8 (24.2)	25 (75.8)
Female (*n* = 37)	29 (78.4)	8 (21.6)	8 (21.6)	29 (78.4)
	χ^2^ = 0.303 *p*-value = 0.582		χ^2^ = 0.068 *p*-value = 0.794	
**Years of clinical practice**				
< 10 years (*n* = 37)	29 (78.4)	8 (21.6)	9 (24.3)	28 (75.7)
> / = 10 years (*n* = 33)	24 (72.7)	9 (27.3)	7 (21.2)	26 (78.8)
	χ^2^ = 0.303 *p*-value = 0.582		χ^2^ = 0.096 *p*-value = 0.757	
**Number of daily consultations**				
< 15 (*n* = 33)	26 (78.8)	7 (21.2)	5 (15.2)	28 (84.8)
> / = 15 (*n* = 37)	27 (73.0)	10 (27.0)	11(29.7)	26 (70.3)
	χ^2^ = 0.321 *p*-value = 0.571		χ^2^ = 2.102 *p*-value = 0.147	
**Frequency of attending to patients aged 60 years and above with multimorbidity daily**				
Daily (*n* = 20)	18 (90.0)	2 (10.0)	8 (40.0)	12 (60.0)
Not daily (*n* = 50)	35 (70.0)	15 (30.0)	8 (16.0)	42 (84.0)
	χ^2^ = 3.108 *p*-value = 0.122^		χ^2^ = 4.667 *p*-value = 0.031*	
**Frequency of attending to patients aged 60 years and above with polypharmacy (> 5 medications) daily**				
Daily (*n* = 14)	13 (92.9)	1 (7.1)	4 (28.6)	10 (71.4)
Not daily (*n* = 56)	40 (71.4)	16 (28.6)	12(21.4)	44 (78.6)
	χ^2^ = 2.797 *p*-value = 0.162^		χ^2^ = 0.324 *p*-value = 0.723	
**Frequency of deprescribing medications during consultations in daily practice**				
Never (*n* = 8)	3 (37.5)	5 (62.5)	1 (12.5)	7 (87.5)
Daily (*n* = 16)	3 (18.8)	13 (81.2)	6 (37.5)	10 (62.5)
Not daily (*n* = 46)	9(19.6)	37 (80.4)	9(19.6)	37 (80.4)
	χ^2^ = 1.150^ *p* = 0.584^		χ^2^ = 2.482^ *p*-value = 0.318^	
**Awareness of the term “deprescribing”**				
Yes (*n* = 56)	46 (82.1)	10 (17.9)	16 (28.6)	40 (71.4)
No (*n* = 14)	7 (50.0)	7 (50.0)	0 (0.00)	14 (100)
	χ^2^ = 6.293 *p*-value = 0.012*		χ^2^ = 5.185 *p*-value = 0.029*	
**Previous training on deprescribing**				
Yes (*n* = 15)	13 (86.7)	2 (13.3)	9 (60.0)	6 (40.0)
No (*n* = 55)	40 (72.7)	15 (27.3)	7 (12.7)	48 (87.3)
	χ^2^ = 1.245 *p*-value = 0.330^		χ^2^ = 14.937 *p*-value = 0.000*	
**Knowledge of deprescribing tool**				
Yes (*n* = 16)	13 (81.2)	3(18.8)		
No (*n* = 54)	40(4.1)	14(25.9)		
	χ^2^ = 0.346 *p*-value = 0.744^			

χ^2=^ Chi square test ^ = fisher’s exact test * = significant *p*-value G

**Fig. 1:**
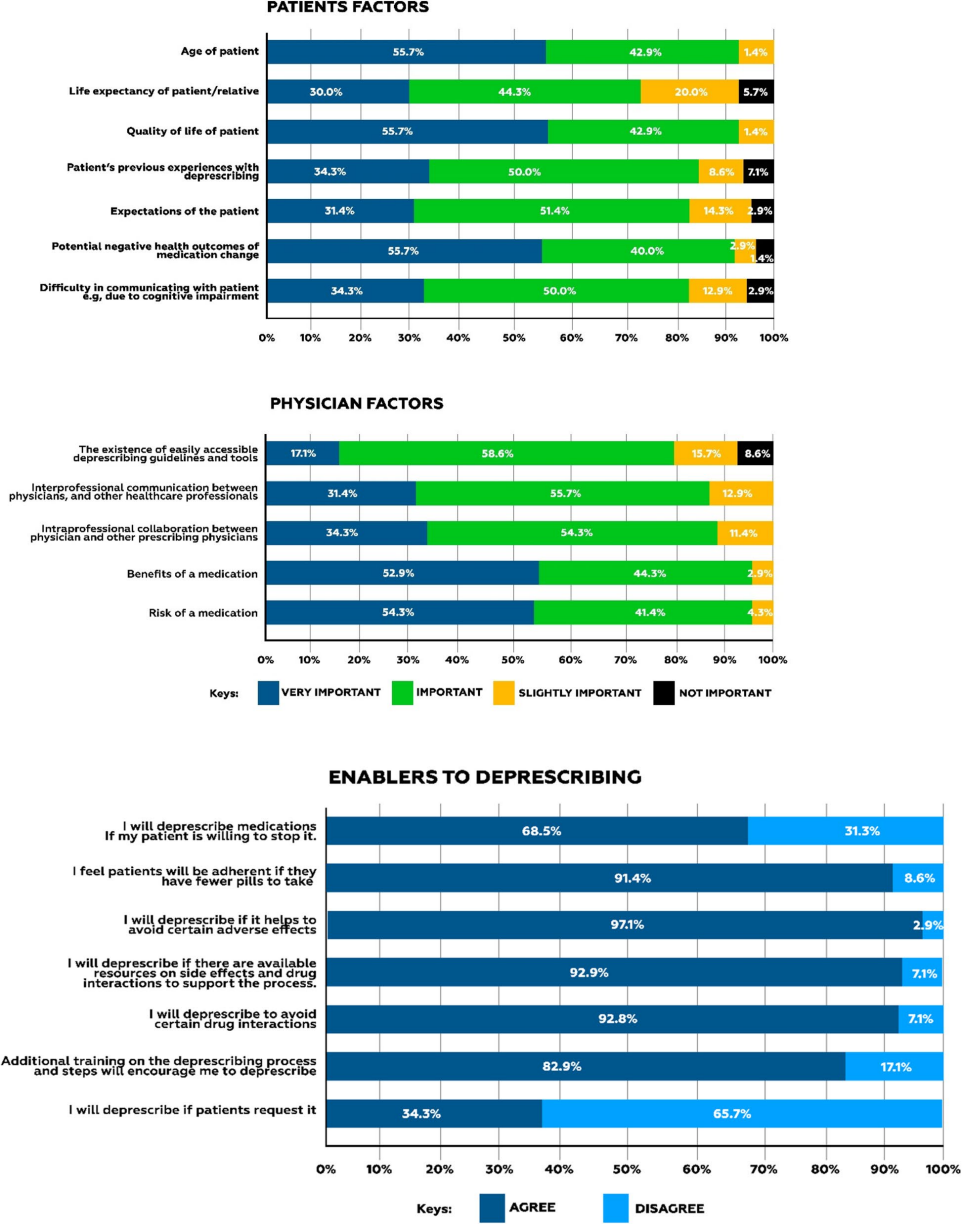
Factors that are important to physicians when making deprescribing decisions

The importance ascribed to some factors that may influence physicians deprescribing decisions was highlighted, most reported enabler to deprescribing is avoidance of adverse effects. Details in Fig. 1.

Physicians also cited other factors that may influence their deprescribing practices. They include; affordability of medications, patient comorbid conditions, culture and socio-economic status of the patient, inadequate time for patient counseling, intersectional communication across medical disciplines and specialties, lack of awareness on deprescribing, availability of medications, medication intolerance, non-availability of drug formularies in counseling rooms, clinical outcome measurements and patients other medications including nutraceuticals. See Fig. 1.

**Fig. 2:**
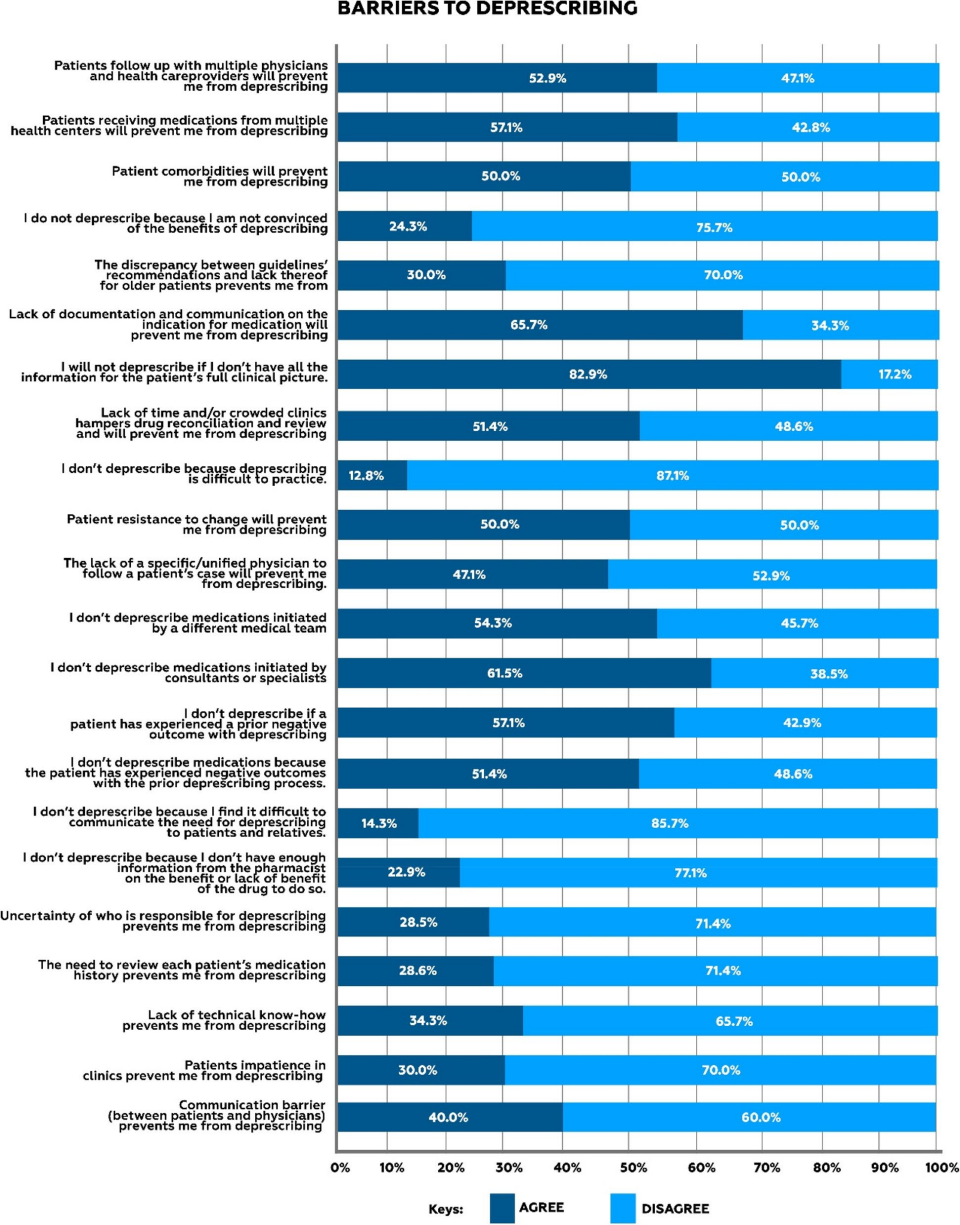
Physicians reported barriers to deprescribing

Various barriers to deprescribing were reported by the physicians such as, Lack of information to patients’ full clinical picture, lack of documentation and communication on the indication for medication, patients’ resistance to change and patients experienced negative outcomes with prior deprescribing process. Details in Fig. 2.

## Discussion

The complexity of caring for older adults, especially in the presence of multimorbidity and polypharmacy highlights the need for deprescribing. While deprescribing is important and may be of great benefit to older multimorbid adults, it is also imperative to explore physicians’ knowledge of the deprescribing process and their perspectives on factors they consider important to the deprescribing process.

Patient related factors important to physician for deprescribing in this study, include; the age of the patient, quality of life of the patient, patient’s previous experiences with deprescribing, patients expectations, negative health outcome of medication change and difficulty communicating with the patient while the physician-related factors reported being important and very important include; intraprofessional communication between physicians, interprofessional collaboration between healthcare workers, and risks/benefits of medications. These are in line with factors cited by other physicians in related literature [11, 23, 25]. It is worthy to note that the physicians acknowledge interprofessional collaboration importance, a pharmacist involvement in the medication use review of patients is highly essential for medication optimization.

There is a good awareness of the term deprescribing (80%) and a significant association between this awareness and knowledge of the deprescribing steps, similar findings have been previously reported by Scott et al. [15]. There was a significant association between knowledge of specific deprescribing tools and awareness of deprescribing, and previous training on deprescribing. Despite this association, less than a quarter of the physicians reported having previous training on deprescribing and were aware of specific deprescribing tools. This indicates that although there exist several evidence-based deprescribing tools and guidelines, the awareness in this setting is limited and underscores the need for further training and sensitization to raise awareness not just on deprescribing, but also on the available tools and guidelines to facilitate the deprescribing process and improve clinical outcome.

Findings from this study also indicate that physicians who frequently attended to older multimorbid patients had better knowledge of specific deprescribing tools. This could be related to their experiences and the need for the physicians to explore the available deprescribing tools due to their frequent exposure to the patients.

The most reported enablers to deprescribing were avoidance of adverse effects of drugs and availability of resources including resources on adverse effects and drug interactions to support the deprescribing process, which shows a proactive attitude of physicians in this setting towards medication management and reduction of incidences of medication adverse effects and events.

Patients’ request to deprescribe was only scantily reported to be an enabler in this setting. This may be because of information asymmetry as patients do not have all the information on the risks and benefits of their medications barring any adverse effects to request for their medicines to be deprescribed. This finding is similar to previously reported study [26].

The most reported barriers were lack of information on the patient’s full clinical picture and lack of documentation on the indication for a medication. Hence, the need for proper documentation of physician clinical decisions cannot be overemphasized to ensure seamless care in the prevailing health system structure of fragmented care. Patients’ reluctance as a barrier to deprescribing was only scantily reported as opposed to findings from similar studies [17]. This may be because older adults have reported a willingness to stop or cease one or more of their medications if it was recommended by their physician [27, 28].

Limitations of our study include reliance on the physicians’ report of the awareness, knowledge and use of deprescribing tools. Most physicians who participated in this study had years of clinical experience below 15 years, thus limiting the representation of older physicians. Our sample may not reflect the general physician population in terms of knowledge and awareness of deprescribing tools because only physician at the teaching hospital were surveyed. Nonetheless, this study has provided insight into awareness and knowledge of deprescribing, as well as factors associated with deprescribing among physicians. It will also contribute valuable baseline evidence to the literature regarding the use of existing deprescribing tools such as Beers and STOPP/START criteria to reduce polypharmacy and the use of potentially inappropriate medication among the elderly.

## Conclusion

There was a good knowledge of deprescribing and the steps to deprescribing. However, there is a dearth of knowledge on deprescribing tools and guidelines. There is a need for further awareness of deprescribing and physicians will benefit from training on deprescribing tools. Policies should be implemented to ensure utilization of existing deprescribing tools to guide physicians deprescribing decision. Further exploration of factors affecting deprescribing is also needed to facilitate development of strategies to mitigate them to further ensure optimization of older persons’ medication.

## Data Availability

The datasets used and analyzed during the current study are not publicly available due to lack of approval by authors and participants for unbounded publication. The corresponding author can be contacted for more information and data provided on reasonable request.

## Abbreviations

FWACP: Fellow of West African College of Physicians
MBBS: Bachelor of Surgery, Bachelor of Medicine
PPO: Potential Prescribing Omissions
START/STOPP: Screening tool of older persons' prescriptions or the screening tools to alert doctors to right treatment
UCH: University College Hospital
